# Infantile recurrent parotitis: follow up study of five cases and literature review

**DOI:** 10.1016/S1808-8694(15)31259-3

**Published:** 2015-10-20

**Authors:** Ivan Dieb Miziara, Victor Eulalio Sousa Campelo

**Affiliations:** 1 Collaborating Professor, Department of Ophthalmology and Otorhinolaryngology, Medical School, USP, Head Physician, Ambulatory of Otorhinolaryngology, Hospital das Clínicas, FMUSP.; 2 Resident Physician, Division of Clinical ENT, Hospital das Clínicas, FMUSP. Hospital das Clínicas, Medical School, USP.

**Keywords:** recurrent parotitis, sialography, sonography

## Abstract

**R**ecurrent parotitis (RP) is defined as recurrent parotid inflammation, generally associated with non-obstructive sialectasis of the parotid gland. It is a rare condition, and its etiology remains an enigma. **Aim:** The purposes of the present study were (1) to relate the follow up of five RP cases; (2) to examine the role of sialography and ultrasound in diagnosis and follow up; and (3) to make a literature review. **Study design:** series review. **Material and Method:** We reviewed all recurrent parotitis cases from the files of the Otolaryngology Division at University of Sao Paulo, Brazil. The criteria for inclusion were at least two years of evolution and more than one year and a half follow-up in our service. We included five children in the study. Sialograhpy was performed in the first evaluation and sonography was executed annually. Recurrent parotitis showed male predominance, and affected mainly children between the ages of 3 and 6. Frequency of crisis improved with time in all cases. Sialography showed sialectasis aspect in the affected glands and sonographic exams demonstrated hipoechoic and heterogeneous internal echoes. One case showed regression of ultrasound changes after clinical improvement.

## INTRODUCTION

**Figure 1:**
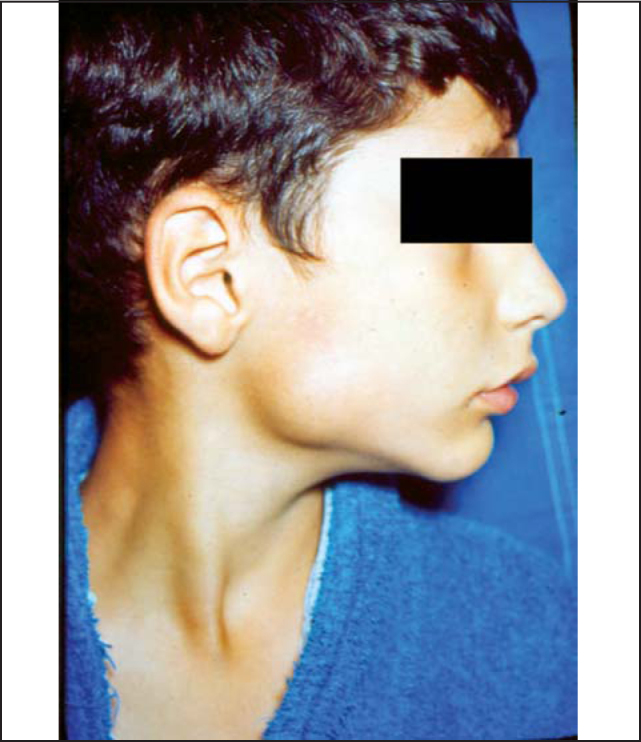
Parotid region bowing.

Recurrent parotitis (RP) is defined as a recurrent inflammation of the parotid gland, normally associated with non-obstructive sialectasis of the gland ^1^. It is also known as juvenile recurrent parotitis, characterized by recurrent episodes of increased volume and/or pain in the parotid gland, normally unilateral (Figure 1), usually followed by fever and general malaise ^2^. There may be discharge of muco-purulent saliva through the duct upon compression of the parotid and many times the volume of secretion is reduced ^3^. The age at onset has been reported as varying from 8 months to 16 years ^4^, and the symptoms normally disappear spontaneously after puberty ^5^.

Recurrent parotitis in children is a well-described condition, but it is rare and of unknown cause. The most widely accepted theories try to solve this affection by reducing the salivary flow that conditions abnormalities in the structure of distal ducts and predispose to recurrent inflammation in the gland ^1^. Other theories try to associate this affection to upper airway infections, allergy, immunodeficiency, autoimmune, congenital sialectasis, however, none of them managed to confirm a real participation in the etiopathogenesis ^1^.

The pathological findings include pseudocystic dilations of interlobular ducts, periductal lymphocytic infiltration, interacinar fibrosis and many degrees of atrophy and fibrosis of acinar glands ^5^.

In most cases of RP, the sialography reveals sialectasis, sometimes combined with ductal affections, The sialogram has been considered the most important and accurate diagnostic test, but there are some particularities that are not desirable, such as exposure to radiation and difficulty to apply it in children. Moreover, there are controversial studies about sialographic findings in the long-term follow-up of these patients. There are no clear criteria to define when a patient needs to be followed up or when he is in recovery ^6^. Recent studies have used ultrasound as an easy to access and non-invasive method to assess salivary glands 5, 6, 7. The ultrasound normally shows hypoechoic areas that correspond to sialectasis demonstrated in sialography ^1^. However, there is still little information about the use of ultrasound in the diagnosis and follow-up of patients with RP 1, 5, 6.

Thus, the present study aimed at: 1) describing the progression of five cases of RP with long-term follow-up; 2) examine ultrasound and sialographic findings of these parameters; and 3) perform literature review about the topic.

## MATERIAL AND METHODS

We reviewed medical charts, sialographies and ultrasounds of children seen in the Division of Clinical Otorhinolaryngology, Hospital das Clínicas, FMUSP, who presented typical clinical findings of recurrent parotitis. Parents and responsible people signed the informed consent and the study was approved by the Ethics Committee, HCFMUSP. We selected all patients that had had at least 2 years of progression of the disease and more than 1.5 year of follow-up. These patients were assessed at least once every 3 months in the first and second year of follow-up and at least once every 6 months in subsequent years. Five children complied with the inclusion criteria. These patients were aged 6 to 16 years and were all male. They were submitted to sialography of parotid gland on the affected sides and bilateral ultrasound at the beginning of the follow-up, in addition to annual ultrasound. Moreover, we performed complete blood count, serum immunoglobulin assays, autoantibodies and inflammatory proteins.

## CASE DESCRIPTION

### Case 1

FDO, 16 years, male, Caucasian, born and resident in Sao Paulo-SP, presented episodes of pain in the left parotid region and had been asymptomatic for at least one year (Table 1). He has been followed up for 5 years and in the admission sialography he had microcalcular images close to canalith extremities, microdiverticuli, terminal ductal ectasia, compatible with chronic sialodenitis. We found heterogeneous texture with hypoechogenic areas in the first four annual ultrasounds of the left parotid region (Figure 2A), in addition to increased intraparotid lymph nodes in the first two exams. These abnormalities were not evidenced in the last ultrasound, performed after 8 years without symptoms, which showed gland within the normal range (Figure 2B). The contralateral parotid gland presented normal aspect in the first ultrasound, heterogeneous architecture in the second and normal pattern in the two last ones. The assays for serum immunoglobulins, autoantibodies and inflammatory proteins were normal. The complete blood count revealed hypochromic and microcytic anemia with Hb 11.4 g/dl. The patient received low-dose corticoids during the crises with maintenance of treatment for four to six weeks. In some crises, the patient used non-hormonal antiinflammatory drugs.

**Table 1:** Clinical progression of patients with recurrent parotitis.

CASES	Age	Age at onset	Duration of follow-up (in years)	Time of progression (in years)	Number of crises	1st year	2nd year	3rd year	4th year	5th year	6th year	7th year	Time without crises (in years)
CASE 1	16	9	5	7	24	6	6	2	3	4	2	1	1
CASE 2	10	3	6	7	8	3	3	2	0	0	0	0	3
CASE 3	8	3	2,5	5,5	11	3	2	2	2	1	1	-	1
CASE 4	9	3	2,5	5,5	23	8	7	7	1	0	0	-	2
CASE 5	7	6	1,5	2	9	5	4	-	-	-	-	-	1/4

**Figure 2A:**
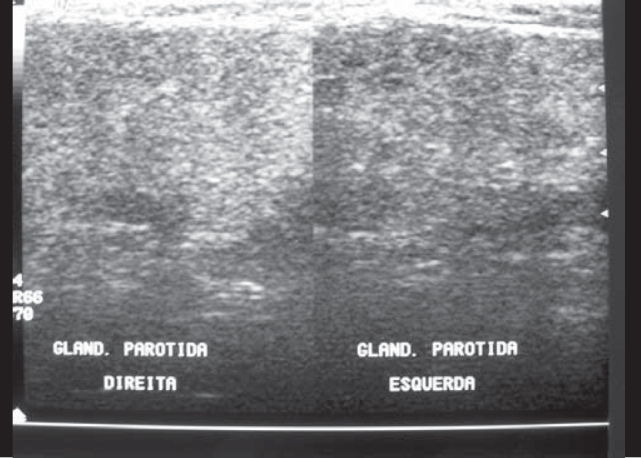
FDO Ultrasound (case 1). Initial examination. Left gland presented heterogeneous texture and the right gland was unaltered.

**Figure 2B:**
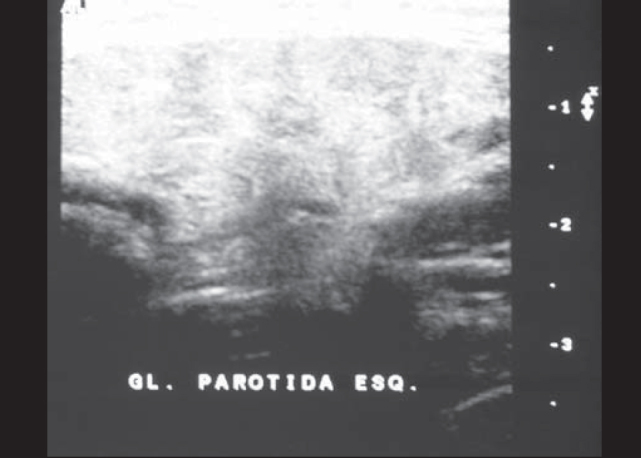
FDO Ultrasound (Case 1): Six years of progression, normal pattern.

### Case 2

MVR, 10 years, male, Caucasian, born and resident in Sao Paulo-SP, presented pain crises in the left parotid region, fever and local bowing since the age of three years, in addition to drainage of purulent secretion through the left parotid gland duct in the first crises. He progressed with a total of 8 crises and had been asymptomatic for approximately 3 years (Table 1). The patient has been followed up for 6 years and the admission sialography showed multiple microdiverticuli in the gland periphery, conveying the aspect of bunch of grapes, suggestive of chronic sialodenitis. He underwent two ultrasounds, one in the first and one in the third year of follow-up. In the first one, we found heterogeneous texture, hypoechogenic areas and increased intraparotid lymph nodes in both parotid glands and increased volume in the left gland. We observed similar findings in the second ultrasound, but intraparotid lymph node increase was not found. The laboratory tests showed IgA deficiency, increase in IgC and IgE levels, increased ASLO and alpha-2 microglobulin. He was treated with low dose corticoids during the crises, maintained for a period of 4 to 6 weeks.

### Case 3

WFA, 8 years, male, Caucasian, born and resident in Sao Paulo-SP, presented episodes of pain and bowing in the left parotid region and fever since the age of 3 years. He had a crisis at the age of 7, from which he progressed with formation of intraparotid abscess that required surgical drainage and antibiotic therapy. The patient has been followed in our center for 2.6 years and has remained asymptomatic for approximately one year after a total of 11 crises (Table 1). The admission sialography showed multiple microdiverticuli on the left gland periphery, conveying the aspect of bunch of grapes, compatible with chronic sialodenitis. He has undergone four ultrasounds that showed heterogeneous texture of left parotid gland with hypoechogenic areas and increased submandibular lymph nodes. The right parotid gland has shown normal aspect in the ultrasound. We did not observe affections to assays with serum immunoglobulins, autoantibodies and inflammatory proteins. The patient has been treated with corticoid in a regimen similar to that used in the above reported cases.

### Case 4

**Figure 3:**
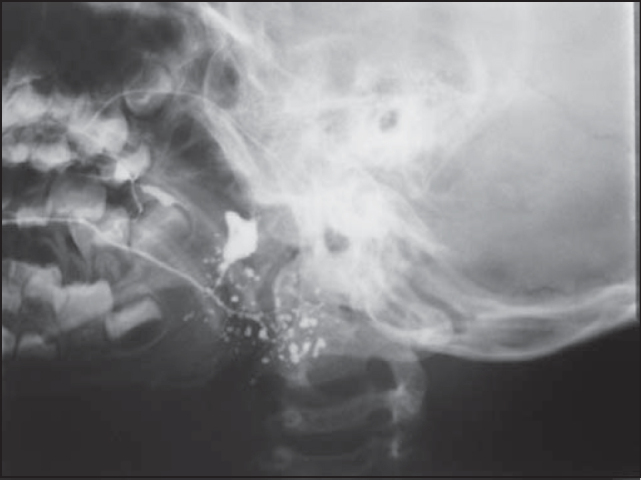
RRL Sialography (case 4) with sacculus dilations in distal ramifications of left Stenon duct, compatible with chronic sialodenitis.

RRL, 9 years, male, Black, born and resident in Sao Paulo-SP, presented with 23 episodes of pain in the left parotid region, fever and local bowing since the age of three years and he has been asymptomatic for approximately 2 years (Table 1). He has been followed up for 3 years and admission sialography showed saccular diffuse dilations in the distal ramifications of Stenon duct on the left, suggestive of chronic sialodenitis and right gland with no affections (Figure 3). He underwent only one ultrasound in the first year, which showed heterogeneous texture of left parotid gland with hypoechogenic areas and increased submandibular lymph nodes. The right parotid gland showed ultrasound pattern within the normal range. The levels of serum immunoglobulins, autoantibodies and inflammatory proteins were within the normal range. The complete blood count revealed a clinical picture of hypochromic and microcytic anemia with 10.4 g/dl. In such patient, we also used low dose corticoids.

### Case 5

MSF, 7 years, male, born and resident in Sao Paulo, presented episodes of pain and bowing in both parotid glands, associated with fever since the age of 6 years. He progressed with a total of nine crises and remained asymptomatic for approximately 3 months (Table 1). He has been followed up in our service for 1,6 year and admission sialography and another one performed in the second year of follow-up showed multiple microdiverticuli on the periphery of the parotid glands, providing an aspect of bunch of grapes, compatible with bilateral chronic sialodenitis. He performed two ultrasounds that showed parotids with reduced dimension, heterogeneous texture with many different cystic images measuring between 3 and 7mm. Serum immunoglobulins, autoantibodies and inflammatory proteins were maintained within the normal range. We observed hypochromic and microcytic anemia with Hb 11.7 g/dl. We followed the same therapeutic regimen with corticoids used in the other cases of RP.

The detailed description of the episodes of flare and follow up of these paranasal sinuses can be found in Table 1.

## DISCUSSION

The onset of RP normally ranges from 3 to 6 years, but there are described cases of earlier or later onset ^1^. Ericson et al., in a study with 20 children, observed onset of cases between three and 16 years ^2^. We found similar pattern in our sample, because out of the five patients in the study, only one had onset of RP after the age of 6 years, developing the first episodes at 9 years. Three patients in our study presented initial symptoms at the age of 3 and the others, at 6 years.

In our study, we found only male patients, which corresponds to the trend in most studies that have demonstrated gender distribution favoring the male gender 1, 8. In a series of 25 patients with RP described by Geterud et al. ^1^, 72% of the patients were male. However, Watkin and Hobsley found a study with 68 patients with RP, out of which 26 were children, with gender distribution similar to childhood, with higher frequency in women than in men (7.5:1) in patients that had onset of the disease after the age of 16 years ^9^.

The number of inflammatory acute crises ranged from individual to individual, with episodes every three or four months, which was the most common pattern 1, 10. Mandel and Kaynar reported that crises tended to occur one to five times a year ^8^. The frequency of episodes is normally higher in the first school year and tends to gradually reduce up to puberty. After puberty, symptoms are usually scarce and vanish completely 1, 10. In our sample, we found increase in number of crises in the two first years of progression, with frequency of 2 and 8 episodes per year in the period, mean of 4.8 crises. We also found a trend of reduction of frequency after the third year of progression.

Galili and Yitczhak proposed two possible mechanisms for spontaneous resolution of the symptoms: total atrophy with consequent absence of symptoms, or regeneration of gland from surviving ductal cells ^1^. Most of the authors favored regeneration as a preponderant mechanism. However, there are persistent cases 1, 10, 11. In our sample, maintenance of the gland architecture after end of the crises period suggests the hypothesis of regeneration of gland tissue.

The clinical picture normally corresponds to painful local swelling associated with fever, which is equivalent to what we found in this study. In two patients, we observed episode of drainage with purulent secretion through the ostium of the parotid duct and another one presented a crisis that complicated with the formation of intraparotid abscess, with surgical drainage need. Most authors reported absence of purulent secretion in their cases^10^, however, Geterud et al. reported drainage of purulent secretion through the duct after compression of the parotid gland ^1^. Swelling normally lasts from some days to two weeks and is spontaneously solved, regardless of the treatment ^8^. In our sample, we decided for treatment of acute crises with low dose corticoids.

The most frequently used test for the diagnosis of RP is still sialography, however, ultrasound has been increasingly used for diagnosis and follow-up as well.

Ultrasound exams performed in the study revealed presence of heterogeneous aspect in 100% of the cases, hypoechoic areas in 40% and intraparotid lymph nodes in 60% of the cases in the initial test. Mayumi et al., assessed 21 glands with RP and found heterogeneous aspect in 80% and hypoechoic areas in 62% ^6^. Rubaltelli et al. observed hypoechoic areas in four out of 10 cases ^5^. Nozaki et al. reported hypoechoic areas in 10 out of 12 glands (83.3%) ^6^. The incidence of hypoechoic areas in cases of RP ranges widely. However, the incidence of heterogeneous glands including hypoechoic areas was of approximately 80% in the three studies.

Mayumi et al. ^6^ observed an increase in echo areas in ultrasound and elimination of hypoechoic areas in three glands with improvement of symptoms or without clinical manifestations. Rubaltelli et al. studied the progression of ten patients with ultrasound, observing important affections in four cases, out of which two showed reduction of heterogeneity after 12 and 18 months ^5^. In the same study, the authors recommended the use of ultrasound to follow up patients with RP, because they would be more sensible to abnormalities of the sialography 5, 6. In our sample, we observed a case (Case 1), in which we found ultrasound abnormalities previously described in the four first exams and normal aspect in the fifth exam, performed 12 months after the crises. These findings suggest the hypotheses that ultrasound may show gland regeneration. Another important observation found in our sample was the presence of bilateral ultrasound affections in 3 to 5 patients, and only one case had bilateral symptoms. This information is correlated with the statements of other authors, in which sialectasias were normally bilaterally, even when only one gland was symptomatic ^12^.

Four of our five patients presented microdiverticuli images in sialography, performed at the onset of follow-up and the other showed pattern of diffuse saccular dilations. The results are in accordance with the literature, because the most common sialography pathological finding in children with RP was sialectasis (microdiverticuli), as described by many authors 3, 13, 14, 15. However, the presence of sialectasis is not pathognomonic of this affection. Different causes of sialectasis have been proposed by the literature. Some authors defend that sialectasias are due to rupture of weakened peripheral ducts, with leak of contrast into the interstitial tissue 3, 16, 17. Others stated that sialectasis corresponds to cystic cavities recovered by epithelium 3, 15.

Most children with RP do not present immune deficit, but children with immunodeficiencies, such as Variable Common Immunodeficiency, may sometimes present RP. For this reason it is recommended to follow them up wit serum immunoglobulin measurements ^12^. In our sample, a patient (Case 2) presented IgA deficit. The presence of this immunodeficiency is not correlated with worsening in progression of RP or onset of complications, and there was interruption of crises three years after onset.

## CLOSING REMARKS

Patients with recurrent parotitis demonstrated male gender prevalence and age of onset predominantly from three to six years. The frequency of crises showed trend to reduction of time. Sialography showed affections compatible to sialectasia in all cases and the ultrasound revealed affections to texture, normally with hypoechoic areas. The ultrasound of patients followed up for long time eventually revealed normal aspect, which can suggest that this exam is advantageous for the follow-up of this disease.
